# HBI-8000, HUYABIO Lead Clinical Program, Is a Selective Histone Deacetylase Inhibitor With Therapeutic Benefits in Leukemia and in Solid Tumors

**DOI:** 10.3389/fonc.2021.768685

**Published:** 2022-01-07

**Authors:** Farbod Shojaei, Bob Goodenow, Gloria Lee, Fairooz Kabbinavar, Mireille Gillings

**Affiliations:** HUYABIO International LLC, San Diego, CA, United States

**Keywords:** HBI-8000, HDACs, oncology, clinical, MOA, tumor immunology

## Abstract

HBI-8000 is a small molecule inhibitor of class I HDACs and has been approved for the treatment of PTCL, ATL and, in combination with exemestane, in a subpopulation of breast cancer. Given the roles of HDACs in normal and cancerous cells, there are currently multiple clinical trials, by HUYABIO International, to test the efficacy of HBI-8000 in monotherapy or in combination settings in leukemias and in solid tumors. The current review is focused on the applications of HDACi HBI-8000 in cancer therapy and its potential in combination with DDR agents.

## Introduction

**Figure 1:**
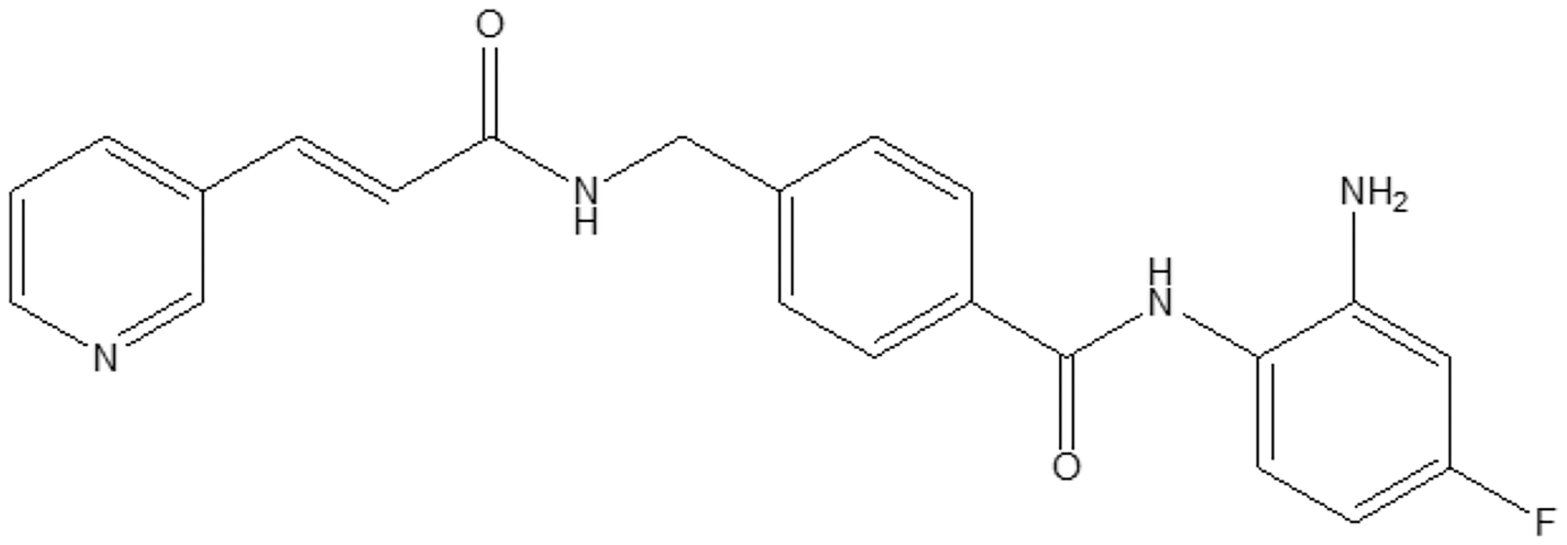
HBI-8000 molecular structure N-(2-Amino-4-fluorophenyl)-4-[N-[(E)-3-(3-pyridyl) acryloyl]aminomethyl] benzamide (C22H19FN4O2; MW 390.41) (4).

HDACs and their role in physiological or disease status have been extensively studied (1). HBI-8000 (also known as CS055, tucidinostat, chidamide, Epidaza^®^ or Hiyasta^®^) is an orally bioavailable small molecule inhibitor of histone deacetylases (HDACs) targeting the catalytic pocket of class I HDACs. Biochemical analysis revealed that HBI-8000 selectively inhibits HDAC1, 2, 3 (class I) and HDAC10 (class II) (2). As an epigenetic modulator, inhibition of HDACs by HBI-8000 affects the expression of multiple downstream genes involved in cancer cell survival and proliferation, thereby suppressing tumor growth and invasiveness (3). HBI-8000 (**Figure 1**) was originally discovered and developed by Chipscreen Biosciences (Shenzhen, China). HBI-8000 showed reasonable safety and tolerability in the IND enabling studies, and was approved in 2014 by the China National Medical Products Administration for the treatment of relapsed/refractory peripheral T cell lymphoma (PTCL) (5). Additionally, HBI-8000 was approved in 2019 in combination with aromatase inhibitors for the treatment of breast cancer (locally advanced or metastatic) (6).

HUYABIO International LLC (HUYABIO, San Diego, CA, USA) successfully licensed the rights to develop and commercialize HBI-8000 globally outside of China. HUYABIO initiated HBI-8000 clinical trials in relapsed/refractory adult T cell lymphoma/leukemia (R/R ATL) in Japan as monotherapy and later in combination with nivolumab in solid tumors in the USA. **Table 1** contains list of all the major clinical trials and indications sponsored by HUYABIO International.

**Table 1:** HBI-8000 clinical trials.

Trial	Phase	Indication	Mono/Combo	Place	Clinical Trial Number	Status
HBI-8000-101	Phase I	All	Mono	USA		Completed
HBI-8000-201	Phase I	NHL	Mono	Japan	NCT02697552	Completed
HBI-8000-210	Phase II	R/R ATL	Mono	Japan	NCT02955589	Completed; JNDA accepted
HBI-8000-203	NDA	PTCL	Mono	Japan	NCT02953652	JNDA submitted
HBI-8000-302	Phase Ib/II	Melanoma, RCC, NSCLC	Combo	USA	NCT02718066	Ongoing, not recruiting
HBI-8000-303	Phase III	Melanoma	Combo	Global (Ex-China)	NCT04674683	Trial initiated
HBI-8000-304	Phase I FE and DDI	Healthy Volunteers	Mono	USA		Completed

NHL, non-Hodgkin’s lymphoma; R/R ATL, relapsed/refractory adult T-cell leukemia/lymphoma; R/R PTCL, relapsed/refractory peripheral T-cell lymphoma; RCC, renal cell carcinoma; NSCLC, non-small cell lung cancer; JNDA, Japan new drug application; FE, food effect; DDI, drug-drug interaction.

## HBI-8000 in Monotherapy

### PTCL in China

PTCL is a subset of non-Hodgkin’s lymphoma (NHL) comprised of the heterogenous populations of T-cells and NK cells (7). There are approximately 50000 patients diagnosed with PTCL in China each year. There is currently no first-line therapy in PTCL mainly due to limited number of patients and lack of randomized clinical trials. The main treatment options include stem cell transplantation and high-dose chemotherapy (5). Recent FDA approval of HDAC inhibitors [romidepsin (8) and belinostat (9)] in USA provided new treatment avenues for R/R PTCL patients. Similarly, chidamide was initially tested in China in R/R PTCL patients and in an open-label, single arm Phase II study with ORR being the primary endpoint (10). Out of 83 patients enrolled in the study, 79 were eligible to receive the therapy based on PTCL diagnosis. Patients were treated with chidamide at 30 mg BIW for 3 weeks and continued to receive the therapy till cancer progression. The ORR was 28% while the median OS and PFS were 21.4 and 2.1 months, respectively [4, 9]. The most notable AEs (≥ grade 3) were neutropenia (11%), leucopenia (13%) and thrombocytopenia (22%). These data led to approval of chidamide as an orphan drug by for R/R PTCL CFDA in 2014 (10).

### R/R ATL in Japan

The safety and efficacy of HBI-8000 in PTCL in China (10) led to HUYABIO-sponsored registration studies in Japan following a Phase 1 trial in NHL (HBI-8000-201). These two registration studies were in PTCL (HBI-8000-203) and in refractory recurrent T-cell lymphoma ATL (HBI-8000-210).

ATL is a cancer of human mature T cells caused by infection with human T-cell lymphotropic virus 1 and is clinically divided into smothering, unfavorable chronic, lymphoma, and acute subtypes (11). According to the data provided by the Japan Ministry of Health, Labor, and Welfare, there are nearly 2000 ATL patients in Japan, 700–1000 of whom die from the disease each year (12). Aggressive ATL has a 3-year survival rate of only 25%. Conventional therapy includes chemotherapy, allogeneic hematopoietic stem cell transplantation, interferon-α treatment, and anti-CC chemokine receptor 4 antibody (mogamulizumab) therapy. The majority of ATL patients, however, develop resistance (relapse or refractory) to the above therapies, further emphasizing the need to develop novel approaches to treat ATL.

In 2016, Hasegawa and colleagues isolated ATL cells from relapsed patients and performed a cell viability assay with HBI-8000. The results of their investigation demonstrated induction of apoptosis in relapsed ATL cells treated with HBI-8000 and with a mean IC50 of 4.35 µM (3). Additionally, treatment with HBI-8000 resulted in the upregulation tumor suppressor genes such as p53 and p21 as an additional mechanism targeting ATL cells (3). Gene expression studies identified the upregulation of Bim, a pro-apoptotic factor, and NLRP3 inflammasomes, confirming the role of HBI-8000 in the induction of apoptosis in ATL cells (3). Additionally, cell cycle analysis in ATL-treated primary cells showed induction of cell cycle arrest in G1 and accumulation of cell in sub-G1 phase indicative of delay in cell cycle progression and potentially cell proliferation (3). When histone acetylation was measured, it was found that exposure to HBI-8000 increased H3 and H4 histone acetylation in the ATL cells, thus confirming HBI-8000 target engagement.

Consistent with the preclinical observations, in a clinical trial twenty-three patients were treated with HBI-8000 orally twice weekly (BIW), ORR was 30.4% [95% confidence interval (CI) 13.2–52.9%]. Median progression free survival (PFS) was 1.7 months (95% CI 0.8–7.4), median duration of response (DOR) was seven months (95% CI 3–9), and median OS was 12.1 months (95% CI 2.1–18.0) (13). All patients experienced adverse events (AEs), predominantly hematologic and gastrointestinal. Incidence of grade ≥3 AEs was 78.3%; most were laboratory abnormalities (decreases in platelets, neutrophils, white blood cells, and anemia). HBI-8000 was well tolerated with expected toxicities that could be managed with supportive care and dose modifications. Results from this clinical trial led to the granting of an orphan disease designation to HBI-8000 for R/R ATL and a marketing approval by PMDA (Pharmaceuticals and Medical Devices Agency) in Japan and under the brand name of Hiyasta^®^.

Additionally, HBI-8000 (chidamide) was approved in China as the treatment for r/r PTCL, under the brand name of Epidaza^®^. In the registration study, and in 79 patients receiving chidamide, the ORR was 28% (22 of 79) including 14% (11 of 79) with complete response/unconfirmed complete response (CR/CRu). Median progression-free survival and overall survival were 2.1 and 21.4 months, respectively. ATL patients tended to have higher ORR (50%) and CR/CRu rate (40%), as well as more durable responses to chidamide treatment. Most adverse events (AEs) were grade 1 or 2, and grade ≥ 3 that occurred in ≥10% patients were thrombocytopenia (22%), leucopenia (13%) and neutropenia (11%), respectively (10). Similarly, in the registration study in patients with r/r PTCL conducted in Japan and in South Korea, in the intent to treatment analysis in a total of 55 patients, the median PFS was 24.1 weeks, median DOR was 50.1 weeks and median OS was 99.1 weeks. Among the 46 patients, evaluable for objective response according to protocol criteria, ORR was 45.7% (21/46 [95% CI: 30.9, 61.0]). ORRs in PTCL subtypes were PTCL-NOS 35.3% (12/34); AITL 87.5% (7/8); ALCL, ALK 33.3% (1/3) and EATL 100.0% (1/1) respectively. All 55 dosed patients experienced adverse events. Most frequent AEs were hematological such as thrombocytopenia and neutropenia, followed by non-hematological AEs such as diarrhea and decreased appetite. AEs led to study drug interruption or dose reduction were observed in 72.7% (40/55) and led to the treatment discontinuation in 32.7% (18/55), respectively. The incidence of Grade ≥3 AEs was 83.6% and most AEs were asymptomatic laboratory abnormalities. HUYABIO submitted the results from this study to PMDA and received regulatory approval in Japan in November 2021.

## HBI-8000 in Combination Therapy

### Combination With Exemestane in HR+ Breast Cancer Patients (ACE Trial)

Preclinical studies indicated that HDAC inhibitors may sensitize resistant breast cancer cell lines to treatment with aromatase inhibitors through reduction of expression and stability of HER2 (14). ERs (estrogen receptor) transcriptional expression is regulated by a balance between recruitment of coactivators (causing transcriptional activation) such as HATs (histone acetyl transferases) (15) vs. recruitment of corepressors (downregulating suppression of transcription) such as HDACs (16). Therefore role of HDAC inhibitors in inhibiting activation of corepressors, leading to continuous expression of ER, suggested a potential therapeutic benefit for HDAC inhibitors in breast cancer in the clinic (17).

In addition to successful trials as monotherapy in subsets of leukemia patients, HBI-8000 was tested in combination with exemestane [steroidal aromatase inhibitor (18)] in post-menopausal HR+ breast cancer patients (6). In a randomized double-blind placebo control Phase III ACE trial, 365 patients were enrolled and assigned to tucidinostat (30 mg BIW) plus exemestane at 25 mg/kg qd (TE; n=244) or placebo (P; n=121) group. Patients were followed up for a median of 13.9 months, PFS was 7.4 vs. 3.8 months in the TE vs. P group respectively, and HR was 0.75 (6). The most common AE (grade 3 or 4) was neutropenia (51% in TE vs. 2% in P), thrombocytopenia (27% in TE vs 2% in P) and leucopenia (19% in TE vs 2% in P). The above data led to CFDA approval of tucidinostat in combination with exemestane in HR+ breast cancer (6).

### Combination With Checkpoint Inhibitors in Solid Tumors

Recently CPIs have established themselves as the mainstay in cancer therapy. They have significantly improved treatment outcome in a subset of cancer patients and demonstrated the role of anti-tumor immunity in tumor progression and aggressiveness (19). However, similar to other anti-cancer agents, patients are either primarily refractory or develop resistance to CPI therapy over the course of treatment. Among the many factors involved in the development of resistance, epigenetic inhibitors have recently attracted increased attention, demonstrating a direct impact on the activity of tumor infiltrating immune cells *via* mechanisms such as i) induction of the activity of antigen-presenting cells and human leukocyte antigen expression, ii) reinvigoration of exhausted T cells, iii) upregulation of the expression of programmed death-ligand 1 (PD-L1) in cancer cells, and iv) modulating Treg cell activity in the tumor microenvironment (20). A recent study by Freeman and colleagues at Harvard University suggested that PD-L1 acetylation status determines its nuclear translocation and is necessary for anti-PD1 activity (21). PD-L1 is initially acetylated by p300 and subsequent deacetylation by HDAC2 determines PD-L1 nuclear translocation. Pharmacologic inhibition of HDAC2 with a selective HDAC2 inhibitor (santacruzamate A), but not of HDAC6, blocks PD-L1 nuclear translocation and induces the expression of immune-related genes involved in boosting anti-tumor immunity (21). Consistently, targeting HDAC2 using small interference RNA or CRISPR-Cas9 recapitulates the pharmacologic inhibition (21). As mentioned in the introduction, HDAC2 is one of the main targets of HBI-8000, providing further evidence that HDAC2 plays a role in the induction of tumor immunity in the tumor microenvironment. Additionally, p53 acetylation showed to play an important role in PD-1 transcription in cancer cells resulting in their growth inhibition independent of the role of PD-1 in the immune system explaining a synergy between HDAC inhibitors and p53 in tumor growth suppression (22). Therefore, HDAC inhibitors appear to play an important role in targeting solid tumors by induction of tumor immunity and directly by acetylation of key components of cancer cells survival.

We tested the efficacy of HBI-8000 in combination with anti-PD1, anti-PD-L1 or anti-CTLA4 monoclonal antibodies in multiple preclinical tumor models (e.g. MC38, CT26, and A20) to investigate its activity in tumor immunity. Compared to single agent CPI monotherapy, HBI-8000 significantly inhibited tumor growth when combined with the above antibodies (23). Mechanistic analysis of tumors using nanoString gene expression showed upregulation of genes responsible for dendritic cell activity, natural killer cells, and cytotoxic T cells in HBI-8000 monotherapy and in combination groups (23). Interestingly, expression of this group of genes was downregulated in the anti-PD1 monotherapy group, further confirming role of HBI-8000 in the induction of activity of key components of tumor immunity (23). Thus, it appears that HBI-8000 plays an important role in converting the tumor microenvironment from cold (immunosuppressive) to hot when combined with CPIs.

The above observations further motivated HUYABIO to initiate a clinical study (HBI-8000-302) to test the safety and efficacy of HBI-8000 (oral, 30 mg, biweekly) in combination with the standard dose of nivolumab (Opdivo^®^, BMS Pharmaceuticals) in patients with melanoma, non-small cell lung cancer, and renal cell carcinoma. Safety analyses showed that the combination was well tolerated. Furthermore, the efficacy observed in the melanoma patients who were naïve to CPI based therapy was encouraging and showed a PFS of 36.9 months vs. 5.7 months for nivolumab monotherapy using publicly available data (24). Furthermore, the overall objective response rate was 69.4%, with 4% complete response and the disease control rate was 94.4% among 36 patients evaluable for objective response. After a median follow-up of 10.8 months among the 38 patients receiving any amount of HBI-8000, the PFS was 36.9 months based on the intent-to-treat analysis. These observations, albeit preliminary, compared favorably with nivolumab monotherapy in this patient population.

The clinical data from Study HBI-8000-302 corroborated the preclinical findings, consisted with the expected role of HBI-8000 in anti-tumor immunity in melanoma patients. HUYABIO is currently conducting the global Phase III program (HBI-8000-303) in several countries around the world.

## HDACs and DNA Damage Response

Independent of HBI-8000 studies, Miller et al., investigated role of HDACs in DDR (DNA damage response) and showed localization of HDACs 1&2 at DNA damage sites causing a reduction in acetylated H3K56 and H4K16 in cell lines treated with laser micro-irradiation (25). Treatment with an HDACi (sodium butyrate) blocked localization of HDAC 1&2 at the DNA damage sites. Depletion of both HDACs 1&2 but not HDAC3 resulted in hyperacetylation of H3K56 and H4K16 in human cancer cell lines. The HDAC 1&2 depleted cells were more sensitive to DDR signaling as measured by γH2AX and showed significant defect in repair mechanisms (26). Overall, these investigations indicated a significant role for HDACs 1&2 in DDR and suggested an additional mechanism for anti-tumor activity of inhibitors of HDAC 1&2 such as HBI-8000. Furthermore, combination of Vorinostat [a pan HDAC inhibitor (27)] and AZD1775 [targeting Wee1, a cell cycle checkpoint molecule (28)] showed synergy in tumor growth inhibition in a preclinical model of AML and *via* induction of DNA damage and premature entry into mitosis (29). These data further indicate an important role for HDAC inhibitors in future combination therapies with DNA damage agents in certain subset of cancer patients.

## Discussion

Epigenetics plays an important role in regulating gene expression in normal and cancerous cells. Epigenetic factors (such as HDACs and DNMTs) have long been studied for their role in tumor progression. Given the ubiquitous expression of epigenetic factors in many cell types in TME and pending cancer type, epigenetic modulators may affect tumor growth *via* several mechanisms mainly induction of apoptosis, promotion of tumor immunity and interference with DDR. Overall, available preclinical and clinical data suggest HBI-8000 play significant roles in cancer therapy, either as monotherapy in ATL and PTCL or in combination with mainstay treatment, such as immune CPIs or aromatase inhibitors, in solid tumors. Recent studies on detailed molecular mechanisms of epigenetic factors along with biomarker identification for patient stratification may support application of epigenetic modulators such as HBI-8000 in combination therapy with other anti-cancer modalities.

## Authors Contributions

FS wrote the first of the manuscript, all authors (FS, BG, GL, FK, and MG) contributed in editing and revising the manuscript in preparation to generate the final version.

## Conflict of Interest

All authors were employed by HUYABIO International LLC.

## Publisher’s Note

All claims expressed in this article are solely those of the authors and do not necessarily represent those of their affiliated organizations, or those of the publisher, the editors and the reviewers. Any product that may be evaluated in this article, or claim that may be made by its manufacturer, is not guaranteed or endorsed by the publisher.

## Abbreviations

AE, Adverse event; AITL, Angioimmunoblastic T-cell lymphoma; ALCL, Anaplastic large cell lymphoma; ALK, Anaplastic lymphoma kinase; AML, Acute myeloid leukemia; ATL, Adult T cell lymphoma/leukemia; CCR4 CC, chemokine receptor 4; CFDA, China Food and Drug Administration; CPI, Checkpoint inhibitor; CI, Confidence interval; DDR, DNA Damage response; DNMT, DNA methyl transferase; EATL, Enteropathy-associated T-cell lymphoma; ER, Estrogen receptor; HR, Hormone receptor; NLRP3, Nucleotide-binding oligomerization domain, leucine rich repeat and pyrin domain containing 3; NSCLC, Non-small cell lung cancer; ORR, Objective response rate; OS, Overall survival; P, Placebo; PFS, Progression-free survival; PTCL, Peripheral T cell lymphoma; PTCL, NOS PTCL Not otherwise specified; R/R, Relapsed or refractory; RCC, Renal cell carcinoma; TE, Tucidinostat plus exemestane.
